# Effects of Structural Isomers of Spermine on the Higher-Order Structure of DNA and Gene Expression

**DOI:** 10.3390/ijms22052355

**Published:** 2021-02-26

**Authors:** Tomoki Kitagawa, Takashi Nishio, Yuko Yoshikawa, Naoki Umezawa, Tsunehiko Higuchi, Chwen-Yang Shew, Takahiro Kenmotsu, Kenichi Yoshikawa

**Affiliations:** 1 Graduate School of Life and Medical Sciences, Doshisha University, Kyoto 610-0394, Japan; true.north.t@gmail.com (T.K.); i.am.angel7329@gmail.com (T.N.); yoshi2989r@gmail.com (Y.Y.); 2 Graduate School of Pharmaceutical Sciences, Nagoya City University, Nagoya 467-8603, Japan; umezawa@phar.nagoya-cu.ac.jp (N.U.); higuchi@phar.nagoya-cu.ac.jp (T.H.); 3 Doctoral Program in Chemistry, The Graduate Center of the City University of New York, New York, NY 10016, USA; shewcy@gmail.com; 4 Department of Chemistry, College of Staten Island, Staten Island, New York, NY 10314, USA; 5 Center for Integrative Medicine and Physics, Institute for Advanced Study, Kyoto University, Kyoto 606-8501, Japan

**Keywords:** polyamine, higher-order structure of DNA, gene expression, bimodal effect of promotion and inhibition

## Abstract

Polyamines are involved in various biological functions, including cell proliferation, differentiation, gene regulation, etc. Recently, it was found that polyamines exhibit biphasic effects on gene expression: promotion and inhibition at low and high concentrations, respectively. Here, we compared the effects of three naturally occurring tetravalent polyamines, spermine (SPM), thermospermine (TSPM), and *N*^4^-aminopropylspermidine (BSPD). Based on the single DNA observation with fluorescence microscopy together with measurements by atomic force microscopy revealed that these polyamines induce shrinkage and then compaction of DNA molecules, at low and high concentrations, respectively. We also performed the observation to evaluate the effects of these polyamine isomers on the activity of gene expression by adapting a cell-free luciferase assay. Interestingly, the potency of their effects on the DNA conformation and also on the inhibition of gene expression activity indicates the highest for TSPM among spermine isomers. A numerical evaluation of the strength of the interaction of these polyamines with negatively charged double-strand DNA revealed that this ordering of the potency corresponds to the order of the strength of the attractive interaction between phosphate groups of DNA and positively charged amino groups of the polyamines.

## 1. Introduction

Polyamines are small multivalent cations that exist in all living organisms. It is well known that polyamines play important roles in many biological functions, such as cell proliferation, differentiation, apoptosis, and gene regulation [1,2,3,4,5,6,7]. They interact with negatively charged macromolecules such as DNA, RNA, and proteins, and thereby regulate the structure and function of these macromolecules [3,8,9,10,11,12,13,14,15,16]. Polyamines induced the compaction/condensation of DNA [17,18,19,20,21], where higher-valence polyamines cause the folding transition at lower concentrations [22,23]. The geometrical arrangement of positively charged amino groups in polyamines also influences their potency for inducing DNA compaction [11,24,25,26]. Recently, it was reported that the change in the conformation of DNA induced by polyamines causes a change in genetic activity through a cell-free gene expression assay [3,27,28,29,30]. It was found that polyamines exhibit biphasic effects on gene expression: promotion and inhibition at low and high concentrations, respectively [31]. This promotion is attributable to the specific shrunken conformation of DNA, which is characterized by parallel ordering among the neighboring DNA segments. On the other hand, inhibition of gene expression is due to the formation of a tightly packed compact arrangement in the higher-order structure of DNA at higher concentrations of polyamines.

**Scheme 1:**
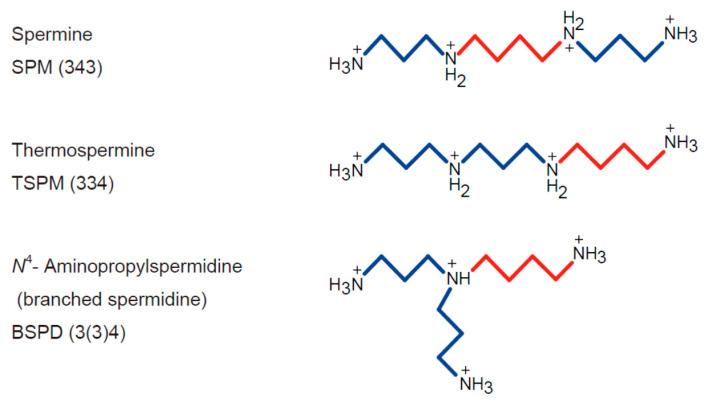
Structural isomers of spermine (4+) examined in this study. Blue and red lines indicate the hydrocarbon chains consisting of three and four carbon atoms, respectively.

In the present study, we investigated the effects of three naturally occurring structural isomers of tetravalent polyamines (spermine (SPM (343)), thermospermine (TSPM (334)), and *N*^4^-aminopropylspermidine (BSPD (3(3)4)) (Scheme 1)) on the conformation and function of DNA. SPM is present in most animals, fungi, seed plants, and some algae, but not in other algae or mosses. TSPM is widely distributed in plants [32,33,34,35], where it is an important factor in controlling xylem differentiation [36], and is also found in some bacteria. BSPD is characterized by a branched-chain structure. It is found in a hyperthermophilic archaeon organism [37,38,39,40] and is thought to help stabilize double-strand DNA at high temperature near the boiling point [41]. Note that the three tetravalent polyamines—SPM, TSPM and BSPD—are produced in hyperthermophilic organisms with different compositions depending on their living environment [37,38,39,40,42]. Archaeal lineage including hyperthermophilic organisms has been regarded as the fundamental system to concern with “origin of life” on the earth [43]. A comparative study among these polyamines as in the present study is expected to contribute to one of the most essential problems in biological science.

## 2. Results and Discussion

### 2.1. Change in The Higher-Order Structure of DNA under Single Molecule Observation with Fluorescence Microscopy (FM)

**Figure 1:**
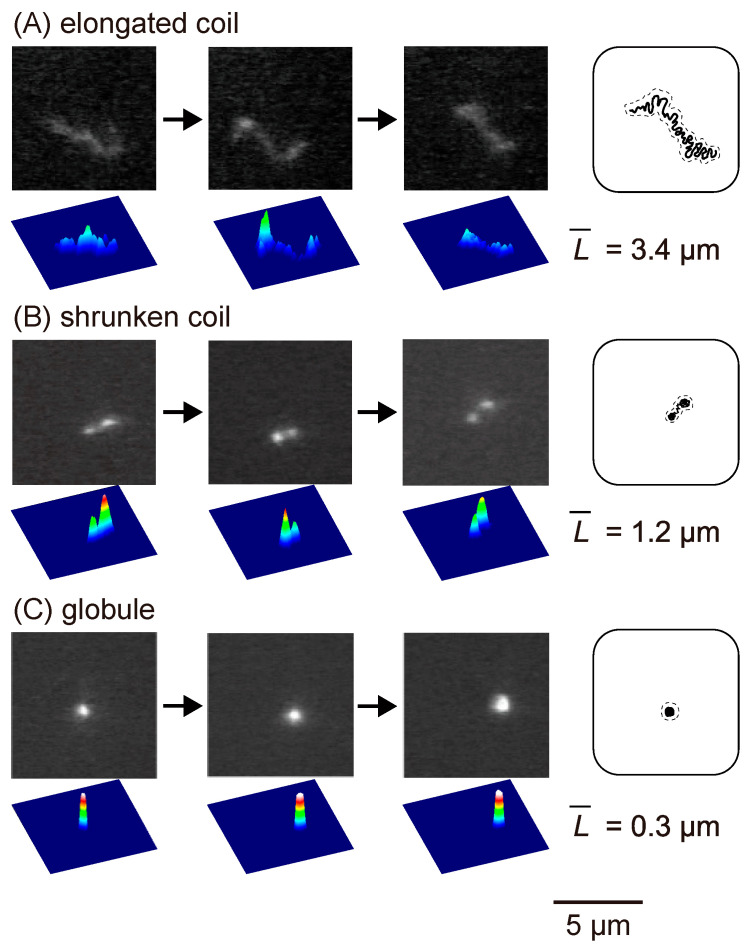
Examples of the fluorescence microscopic (FM) images of single T4 GT7 DNA molecules in bulk solution exhibiting Brownian motion. Corresponding quasi-3D profiles on the spatial fluorescence intensity distribution are also shown. The total observation time is 1 second. (**A**) In the absence of polyamines, (**B**) 50 µM SPM, and (**C**) 80 µM SPM. The top row shows fluorescence microscopic images. $\bar{L}$ is the time-average of the long-axis length. A blurring effect on the order of 0.3 µm is observed for the FM images.

**Figure 2:**
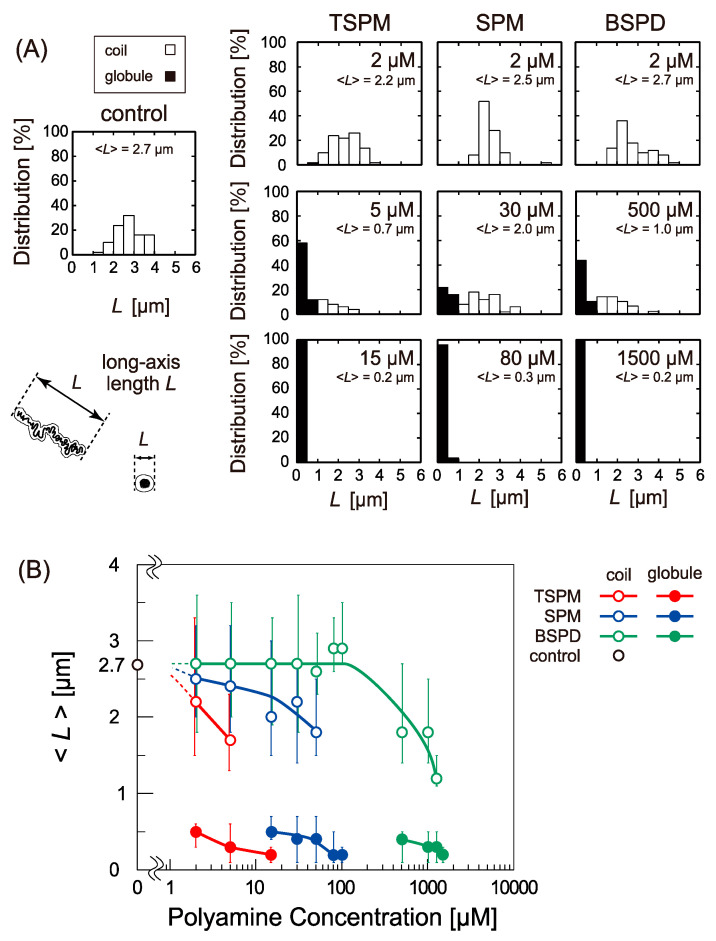
(**A**) Histograms of the long-axis length *L* of T4 GT7 DNA evaluated through FM observations at different concentrations of the polyamines TSPM, SPM, and BSPD. For each condition, 50 DNA molecules were measured. The DNA concentration was 0.1 µM in nucleotide units. (**B**) Ensemble average <*L*> of the long-axis length of T4 GT7 DNA. The distribution widths of *L* due to Brownian motion are depicted with thin vertical lines. The populations in the coil and globule states are indicated by white and black bars in the histogram.

Figure 1 displays the real-time imaging of single T4 GT7 DNA (166 kbp) molecules in bulk solution at different concentrations of spermine (SPM). The time-dependent fluorescent images of the DNA conformation provide reliable information regarding whether individual DNA molecules are in a fluctuating coil state or a compact globular state. Based on these FM observations, the distributions of the long-axis length L of DNA were evaluated at different concentrations of the spermine isomers, TSPM, SPM, and BSPD (Figure 2A). In the histogram, the difference between the coil state and compact state was denoted by using white and black bars, suggesting that the coil and globule states coexist for intermediate concentrations of polyamines. As shown in Figure 1, a higher-order structural change on individual DNA molecules under Brownian motion has been captured in solution to avoid the effect of surface adsorption. Despite the relatively lower resolution compared to electron microscopy (EM) and atomic force microscopy (AFM), we have adapted T4 GT7 DNA (the contour length: 57 μm) to illustrate that the DNA with such a length is sufficient to be monitored by FM [21,23,24,25,26,28,29,30,31,32,40,41]. Here, note that FM observation on single DNA molecules provides information of its solution structure, enabling clear distinguishing among elongated coil, compaction, intrachain segregation, aggregation, and precipitation involving single or multiple DNA. Identifying these states of DNA molecules is either highly challenging or almost impossible in the current studies of so-called DNA condensation [8,9,10,11]. Figure 2B shows the variation in the ensemble average of the long-axis length, <L>, depending on the polyamine concentration. The figure plots the values of <L> for the coil and globule states separately. The potential to cause the folding transition is found in the order TSPM > SPM > BSPD, the transition is observed at several µM for TSPM, several tens of µM for SPM, and several hundred µM for BSPD, indicating that the relative ratios of the transition concentrations close to 1: 10: 100 for TSPM, SPM, and BSPD.

### 2.2. Activity of Gene Expression

**Figure 3:**
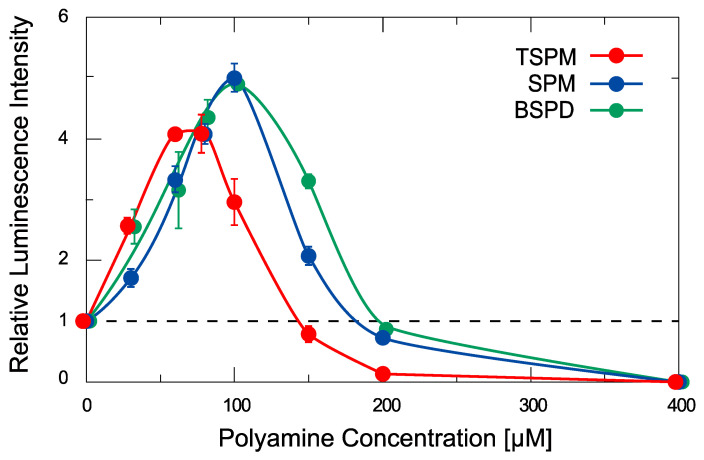
Efficiency of gene expression vs. concentrations of the polyamines TSPM, SPM, and BSPD, as observed from the emission intensity of luciferin–luciferase reaction. The intensity is normalized to the control condition (=1). The DNA concentration was set at 0.3 µM in nucleotide units. The error bar shows the standard deviation.

The effects of the polyamine isomers on the activity of gene expression were measured by using a cell-free luciferase assay. Figure 3 shows the relative luminescence intensity as a marker of gene expression at different polyamine concentrations obtained for 90 min of incubation, where the intensity is normalized to the control experiment observed in the absence of polyamine. All of the isomers exhibit a biphasic effect (promotion followed by inhibition) on gene expression with an increase in their concentration. This biphasic effect on gene expression reproduces recently reported experimental trends observed with the increase of SPM concentrations [31]. Figure 3 demonstrated that TSPM has a greater inhibitory effect than SPM and BSPD, and all of the polyamines exhibit the bimodal effect of promotion and inhibition depending on their concentration.

**Figure 4:**
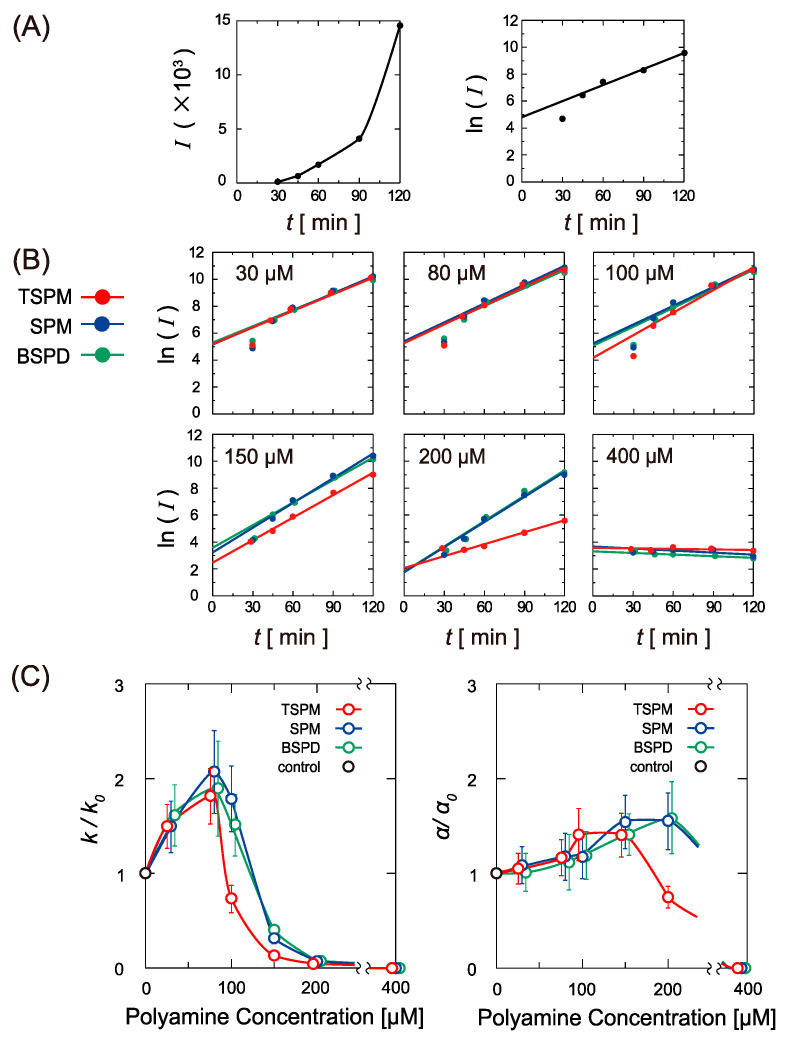
(**A**) Left: Temporal changes in gene expression up to *t* = 120 min in the absence of polyamine, where *I* is the luminescence intensity evaluated through a luminometer. Right: Logarithm of the luminescence intensity, ln(*I*), vs. time, *t*, indicating a nearly linear relationship between *t* = 45 and 120 min. The slope gives the reaction constant with an exponential increase in the product of gene expression. (**B**) Logarithm of the luminescence intensity, ln(*I*), vs. time, *t*, for the experimental data in the presence of different concentrations of the polyamines. (**C**) Relative rate constants, $k/k_{0}$ and $\alpha /\alpha_{0}$, as the pre-exponential and exponential parameters in Equation (1) for gene expression as a function of the polyamine concentration. The rate constants in the absence of the polyamine are $k_{0}$ = 4.9 min^−1^ and $\alpha_{0}$ = 0.039 min^−1^. Error bar was estimated based on the consideration of error propagation product.

To gain further information on the kinetic effect of polyamines on gene expression, we measured the time-dependent change in the luminescence intensity, *I*. Figure 4A shows time-dependent increase in *I* in the absence of polyamine from 30 min until 120 min, indicating a gradual acceleration of the rate of gene expression after a short initial period on the order of 30 min. The logarithmic plot in the right column of Figure 4A actually reveals an exponential increase in *I* vs. time, except for the short initial period. Figure S1 shows the raw data for the time-dependent change in luminescence intensity in the absence of polyamine for the period up to 360 min, where the exponential increase changes to a stationary state for the late stage at around 300–360 min. Figure S2 shows the raw data for the time-dependent change in the luminescence intensity at different concentrations of the polyamines, corresponding to the logarithmic data in Figure 4B. Similar accelerating effects on the kinetics of gene expression for the early period of the reaction have been reported [44,45,46]. If we assume that the rate of transcription increases in an exponential manner, gene expression should proceed as follows:

(1) $$\frac{dx}{dt}=\;k\;\exp\;\left(\alpha t\right)$$

Through integration based on the initial condition, *x* = 0 when *t* = 0,

(2) $$x=\;\frac{k}{\alpha }\;(\exp\;\left(\alpha t\right)-1)$$

When exp(αt)≫1,

(3) $$\ln x\approx \;\alpha t+\ln\left(\frac{k}{\alpha }\right)$$

Based on this argument, it becomes possible to evaluate the kinetic constant *k* from the slope α and cross section $\ln\left(\frac{k}{\alpha }\right)$ on the vertical axis in the logarithmic plots given in Figure 4A,B. In this figure, the luminescence intensity *I* used as a function corresponding to *x* in the above argument with Equations (1)–(3). Figure 4C shows the dependence of the kinetic parameters, $k/k_{0}$ and $\alpha /\alpha_{0}$, on the polyamine concentrations with the three different isomers, where $k_{0}$ and $\alpha_{0}$ are the parameters in the absence of polyamine. The change of $k/k_{0}$ is rather sensitive to the concentration. On the other hand, $\alpha /\alpha_{0}$ varies relatively up to a concentration of 150 µM. Notably, for all three polyamines, both kinetic parameters become almost zero at 400 µM. These results imply that the polyamine isomers mainly change the pre-factor *k* in Equation (1), by keeping similar exponential factors over time. Among the polyamine isomers, the parameter $k/k_{0}$ changes in a similar manner up to 80 µM. Beyond that concentration, TSPM tended to slow the reaction rate at 100 µM, whereas SPM and BSPD maintained a relatively high promoting effect. At higher concentrations, TSPM inhibited gene expression with a higher potency than SPM and BSPD: TSPM > SPM ≥ BSPD. At 400 µM, all three polyamines completely inhibited gene expression.

### 2.3. Atomic Force Microscopy (AFM) Observation

**Figure 5:**
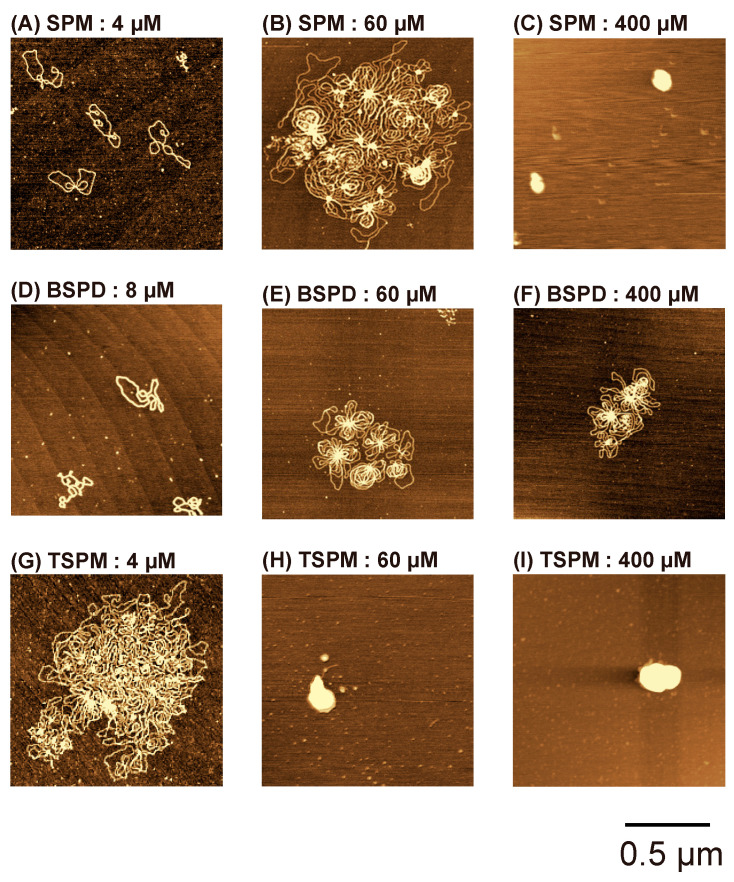
Atomic force microscopy (AFM) images of plasmid DNA (4331 bp) in the presence of various concentrations of (**A**–**C**) SPM, (**D**–**F**) BSPD and (**G**–**I**) TSPM. All specimens were gently adsorbed on a flattened mica surface without shear stress and then dried. The DNA concentration was set at 0.3 µM in nucleotide units.

To clarify the relationship between gene expression and the higher-order structure of DNA, we observed the DNA conformation under treatment with polyamines by AFM. Figure 5 shows typical AFM images of plasmid DNA (4331 bp) in the presence of SPM, TSPM, or BSPD. At 4 µM SPM (Figure 5A) and at 8 µM BSPD (Figure 5D), DNA molecules were separately dispersed in a relaxed state on the mica surface. As for BSPD, it was difficult to run the AFM measurement because of the rather low adhesivity of DNA on the mica surface at 4 µM. Thus, the 8 µM BSPD is adopted in Figure 5D. At 4 µM TSPM (Figure 5G), early condensation of multimolecular DNA formed an aggregate consisting of multiple nodal points. As the concentration of SPM was increased to 60 µM, flower-like complexes formed by the assembly of multiple DNA molecules (Figure 5B). At 400 µM SPM, DNA molecules exhibited a tightly packed conformation (Figure 5C). A similar change in conformation was observed with an increase in BSPD, at 60 µM BSPD, some small flower-like structure was observed (Figure 5E). However, even at 400 µM BSPD, the tightly packed conformation as in Figure 5C was not observed (Figure 5F). This is because BSPD, a branched-chain polyamine, has less potential to induce DNA compaction than the two linear-chain polyamines. On the other hand, TSPM has the highest potential to induce DNA compaction among the three, and forms a tightly packed conformation of DNA at a low polyamine concentration (Figure 5H). The conformation of DNA changed according to the potential of TSPM, SPM, and BSPD to inhibit gene expression. The results of AFM observation support the idea that the highly condensed structure of DNA inhibits gene expression. On the other hand, the appearance of the flower-like conformation at the polyamine concentrations lower than those to induce DNA compaction is expected by considering the activation of gene expression. We have discussed the detailed mechanism of the acceleration of gene expression in the presence of a relatively low concentration of polyamines in our past publication [31]. Note that the polyamine concentration in the AFM observations does not directly correspond to that for the FM observations and also for the measurements in vitro gene expression, i.e., the AFM measurements undertake the DNA absorbed on a solid substrate being different from the FM measurements and luciferin–luciferase reaction.

### 2.4. Numerical Modeling of the Interaction of Polyamines with Double-Strand DNA

The experimental results indicate that TSPM enhances and inhibits the efficiency of gene expression at lower concentrations than SPM and BSPD. This likely means that TSPM interacts more stably with DNA than SPM and BSPD. To shed light on the physicochemical mechanism that underlies the different nature of the interaction of the polyamines with DNA, we performed a numerical study by adapting a simple model of the interaction between DNA and the polyamines, TSPM, SPM and BSPD (modeled as rigid molecules with explicit charged sites), in terms of electrostatic interaction. It is expected that a highly negatively charged DNA molecule is neutralized by addition of the polyamines, which causes a change in the higher-order structure of DNA from the coil to globule states above a certain critical concentration [12,21,23,24,25,26]. In the present work, we adopted the screened Coulomb potential to describe the interaction of the negative charges of phosphate groups in DNA with the positive charges of the polyamines, which is expressed as follows:

(4) $$\begin{array}{ll} \frac{V\left(r\right)}{k_{B}T} & =\infty \;\;\;\;\;\;\;\;\;\;\;r<r_{c}\\ & =\frac{\Gamma q_{i}Q_{j}\exp\left(-\kappa r\right)}{r}\;\;r\geq r_{c} \end{array}$$

where $k_{B}$ is the Boltzmann constant. T is the temperature. r is the separation between a positive charge in a polyamine and a negative charge of a phosphate group in the DNA. $q_{i}$ is the i-th charge in the polyamine. $Q_{j}$ is the j-th negative charge of a phosphate group in DNA. $q_{i}$ and $Q_{j}$ are normalized to unity by an elementary charge. Γ is the interaction strength, and has the value of the Bjerrum length, 0.68 nm, at 25 ℃ in aqueous medium by considering the effect of counter ion condensation. κ is the inverse Debye screening length of 2.4 nm^−1^. $r_{c}$ is approximated to be 0.31 nm, which is a typical closest distance between a charged amine and a phosphate group in DNA. The details of the modeling of the interaction between polyamines and DNA, together with the reason why we chose these values for the parameters, have been reported previously [47].

**Figure 6:**
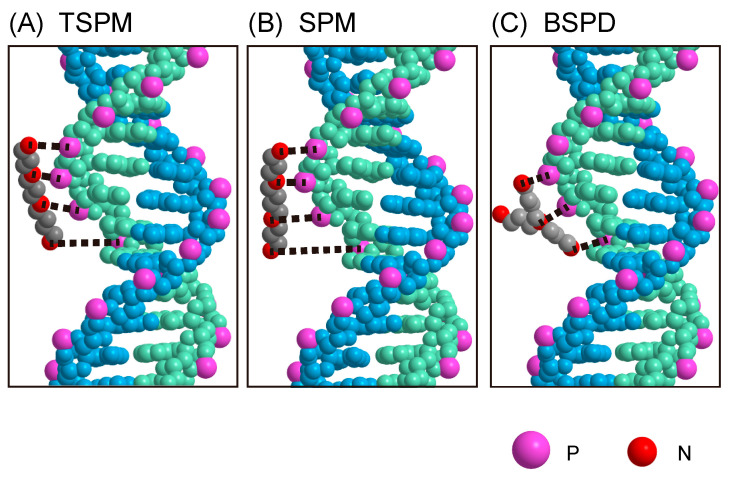
The most stable spatial arrangement of the polyamines interacting with double-stranded DNA, evaluated through a numerical simulation under a simple model in the framework of electrostatic interaction (see the main text). Electrostatic interaction mode was evaluated as the attractive interaction between the negatively charged phosphate groups of DNA and positively charged polyamines. The phosphate groups of DNA are indicated by purple beads, and the amino groups of polyamines are indicated by red beads. The adjacent pairs of negatively charged moieties in the DNA model are separated by 0.34 nm.

Figure 6 shows a schematic diagram of the interaction of the negative charges of a phosphate group in DNA with the positive charges of the polyamines in the framework of electrostatic interaction, where the strongest interaction for each negative charge of DNA is shown by a black dotted line. The polyamines as depicted in Figure 6 are positioned in such a way that their centers are at a closest distance of 0.34 nm from the center of DNA and the helix structure of DNA is used to describe the positions of the negative charges of DNA in the calculations. If we consider the major contributors to the electrostatic interaction of DNA with the polyamines, the numbers of positive and negative charges are taken as follows: $q_{i}$ (i = 1,····,4) for the polyamines and $Q_{j}$ (j = 1,····,5) for the phosphate groups. Based on the results calculated with Equation (4), the energies of electrostatic interaction normalized by thermal energy of $k_{B}T$ for TSPM, SPM and BSPD are 3.87, 3.28 and 3.18, which correspond to 2.31, 1.96 and 1.90 kcal/mol, respectively. The numerical results indicate that TSPM exhibits the strongest binding to DNA in terms of electrostatic interaction among the three polyamines, i.e. the potency exhibits the ordering of TSPM > SPM ≥ BSPD. This means that TSPM neutralizes the negative charge of DNA most efficiently. Thus, this simulation with simple modeling reproduces the essential feature of the different effects of the polyamines on DNA. Based on the calculation results, the electrostatic interaction between DNA and the polyamines plays an important role in the higher-order structure transition and contributes to the activity of gene expression.

## 3. Materials and Methods

### 3.1. Materials

Potassium chloride and an antioxidant, 2-mercaptoethanol (2-ME), were purchased from Wako Pure Chemical Industries (Osaka, Japan). Spermine, SPM (343), was purchased from Nacalai Tesque (Kyoto, Japan). Thermospermine, TSPM (334), was purchased from Santa Cruz Biotechnology (Dallas, TX, USA). *N*^4^-Aminopropylspermidine, BSPD (3(3)4), was synthesized according to a previous report [40]. T4 GT7 bacteriophage DNA was purchased from Nippon Gene (Tokyo, Japan). The fluorescent cyanine dye YOYO-1, (1,10-(4,4,8,8-tetramethyl-4,8-diazaundecamethylene(bis(4-((3-methylbenzo-1,3-oxazol-2-yl) methylidene]-l,4-dihydroquinolinium] tetraiodide) was purchased from Molecular Probes, Inc. (Eugene, OR, USA). Plasmid DNA (Luciferase T7 Control DNA, 4331 bp) containing a firefly luciferase gene and a T7 RNA polymerase promoter sequence was purchased from Promega (Madison, WI, USA). Other chemicals obtained from commercial sources were of analytical grade.

### 3.2. Methods

#### 3.2.1. Direct Observation of the Higher-Order Structure of DNA in Bulk Solution by Fluorescence Microscopy (FM)

T4 GT7 DNA was dissolved in a Tris-HCl buffer (10 mM), 4% (*v/v*) 2-ME, and KCl (80 mM) at pH 7.5 in the presence of various concentrations of polyamines (0–1500 µM). Measurements were conducted at a low DNA concentration (0.1 µM in nucleotide units). To visualize individual DNA molecules by FM, 0.05 µM of YOYO-1 (Excitation/Emission 491/509 nm) was added to the DNA solution. Single-molecule observations were performed with an Axiovert 135 inverted fluorescence microscope (Carl Zeiss, Oberkochen, Germany) equipped with a 100× oil-immersion objective lens and fluorescent illumination from a mercury lamp (100 W) via a filter set (Zeiss-10, excitation BP 450–490; beam splitter FT 510; emission BP 515–565). Images were recorded onto a DVD at 30 frames per second with a high-sensitivity EBCCD (Electron Bombarded Charge-Coupled Device) camera (Hamamatsu Photonics, Shizuoka, Japan) and analyzed with the image-processing software ImageJ (National Institute of Mental Health, Bethesda, MD, USA). Based on the observation of time-successive images, the long-axis length L of each DNA in solution was evaluated. Ensemble averages of the long-axis length <L> for 50 DNA molecules were evaluated at each experimental condition.

#### 3.2.2. Luciferase Assay for Gene Expression

A cell-free luciferase assay with a TnT T7 Quick Coupled Transcription/Translation System (Promega) was carried out according to the manufacturer’s instructions as follows. Plasmid DNA encoding firefly luciferase with a T7 promotor was used as the DNA template. The DNA concentration was 0.3 µM in nucleotide units. The reaction mixture was incubated for 90 min at 30 °C on a Dry Thermo Unit (TAITEC, Saitama, Japan) at each experiment with the desired concentration of an individual polyamine. To evaluate the kinetics of the gene expression, measurements were also performed with different incubation periods between 30 and 120 min. Luciferase expression was evaluated following the addition of luciferase substrate (Luciferase Assay Reagent, Promega) by detecting the emission around 565 nm using a luminometer (MICROTEC Co., Chiba, Japan). We evaluated the intensity of luminescence according to the apparent strength represented with a luminometer by following the manufacturer’s instruction.

#### 3.2.3. AFM Measurements

For AFM imaging using an SPM-9700 (Shimadzu, Kyoto, Japan), 0.3 µM plasmid DNA was dissolved in Tris-HCl (10 mM) buffer solution (pH 7.5) with various concentrations of polyamines. The DNA solution was incubated for more than 10 min and then transferred onto a freshly cleaved mica surface. After it was allowed to stand for 10 min at room temperature (25 °C), the mica was rinsed with ultra-pure water and dried under nitrogen gas. All measurements were performed in air using the tapping mode. The cantilever (OMCL-AC200TS-C3, Olympus, Tokyo, Japan) was 200 µm long with a spring constant of 9–20 N/m. The scanning rate was 0.4–1.0 Hz and images were captured using the height mode. The obtained images were plane-fitted and flattened by the computer program supplied with the imaging module.

## 4. Conclusions

The new observations and essential novelties in the present study are briefly summarized as follows: (1) The tetravalent polyamines cause activation and then induce the complete inhibition in gene expression, depending on their concentration. (2) The complete inhibition of gene expression is attributed to the tight compaction of DNA. (3) The activation of gene expression is generated for the DNA with shrinkage conformation. (4) The kinetics of gene expression suggests the existence of a nonlinear feedback mechanism on the coupled process of transcription and translation. (5) The inhibition efficiency of the polyamines on gene expression exhibits the ordering of TSPM > SPM ≥ BSPD, which is attributable to the difference in how the cationic moieties of the polyamine interact with the negatively charged phosphate groups of double-strand DNA.

It is expected that the structural/conformational study at a single-DNA level through a cooperative study with numerical calculations, as revealed here, would provide useful information for our understandings in numerous unsolved biological problems. In addition, an important challenge in future studies would be to investigate the chemical kinetics underlying gene expression, which is characterized by an exponential or a self-catalytic increase in protein production after an initial lag period. It is of interest hereafter to clarify the effects of polyamines both on transcription and translation in relation to the observed acceleration of gene expression. Especially, a quantitative evaluation of the nonlinear kinetic effect of both transcription and translation is awaited. Extension of the present study would be of scientific value in relation to the fundamental mechanism of living organisms on Earth.

## Data Availability

All data is contained within the article or supplementary material.

## Supplementary Materials

(Original data on the time course of the luminescence intensity given in Figure 4A,B) can be found at https://www.mdpi.com/1422-0067/22/5/2355/s1.

## Abbreviations

SPM	Spermine
TSPM	Thermospermine
BSPD	*N*^4^-Aminopropylspermidine
FM	Fluorescence Microscopy
AFM	Atomic Force Microscopy
