# Supplementary material for: Effects of Structural Isomers of Spermine on the Higher-Order Structure of DNA and Gene Expression
Source: Int J Mol Sci. 2021 Feb 26;22(5):2355. doi: 10.3390/ijms22052355 (PMC7956460; doi:10.3390/ijms22052355)
Supplement: Supplementary file 1 [file ijms-22-02355-s001.pdf]

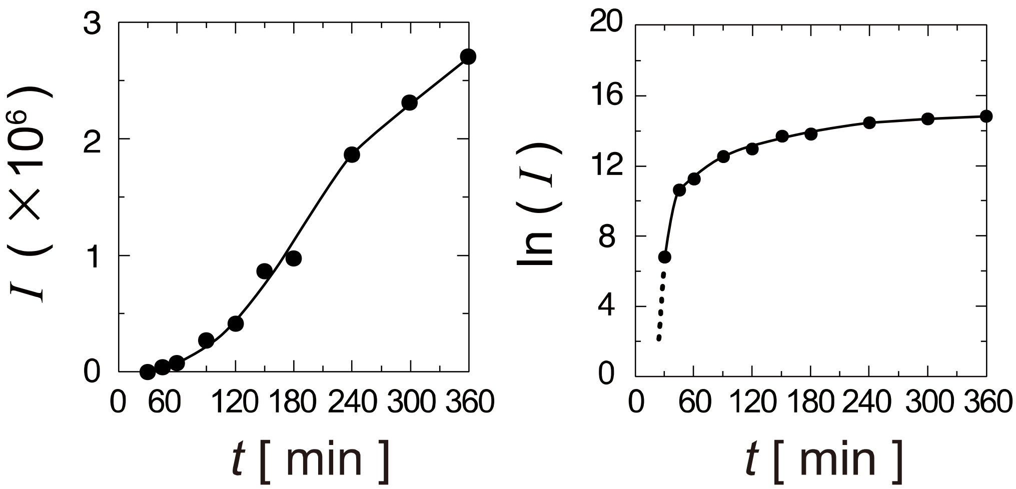

Figure S1. Left: Time-dependent change in the luminescence intensity in the absence of polyamine for the period until 360 min, corresponding to the observation given in Figure 4A. **Right**: Logarithmic plot.

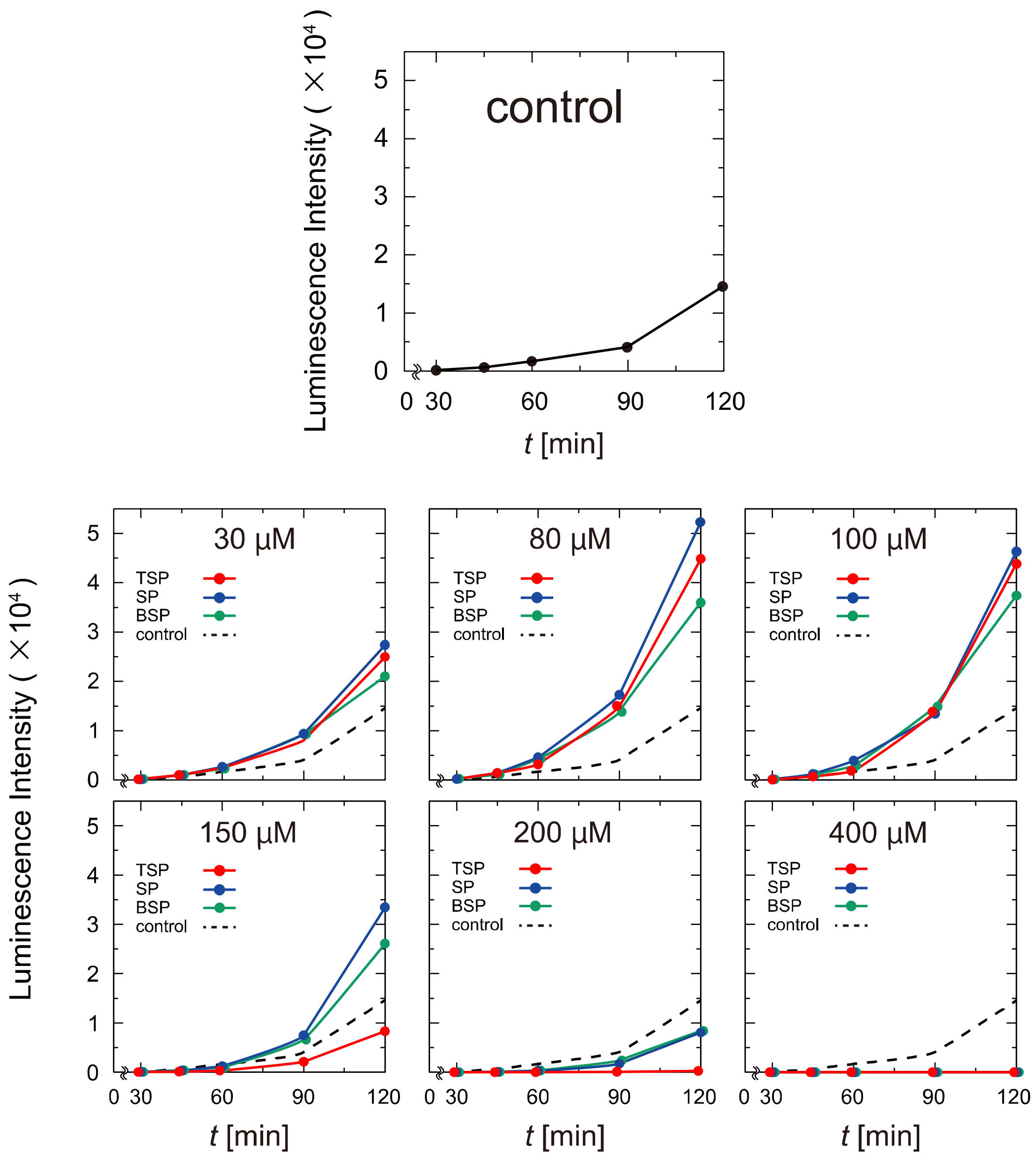

Figure S2. Time-dependent change in the luminescence intensity at different concentrations of the polyamines, corresponding to the logarithmic data given in Figure 4B.
